# An Overview of the Chemical Characteristics, Bioactivity and Achievements Regarding the Therapeutic Usage of Acetogenins from *Annona cherimola* Mill.

**DOI:** 10.3390/molecules26102926

**Published:** 2021-05-14

**Authors:** Alexandra G. Durán, M. Teresa Gutiérrez, Francisco J. R. Mejías, José M. G. Molinillo, Francisco A. Macías

**Affiliations:** Allelopathy Group, Department of Organic Chemistry, Institute of Biomolecules (INBIO), Campus de Excelencia Internacional (ceiA3), School of Science, University of Cadiz, C/República Saharaui, 7, Puerto Real, 11510 Cadiz, Spain; alexandra.garcia@uca.es (A.G.D.); mariateresa.gutierrez@uca.es (M.T.G.); javi.rodriguezmejias@uca.es (F.J.R.M.); chema.gonzalez@uca.es (J.M.G.M.)

**Keywords:** *Annona cherimola*, acetogenins, bioactivity, acetogenin encapsulation, acetogenin formulation, acetogenin drug delivery

## Abstract

*Annona cherimola* Mill., or the custard apple, is one of the species belonging to the Annonaceae family, is widely used in traditional medicine, and has been reported to be a valuable source of bioactive compounds. A unique class of secondary metabolites derived from this family are Annonaceous acetogenins, lipophilic polyketides considered to be amongst the most potent antitumor compounds. This review provides an overview of the chemical diversity, isolation procedures, bioactivity, modes of application and synthetic derivatives of acetogenins from *A. cherimola* Mill.

## 1. Introduction

Plants are one of the most important and diverse sources of chemical structures and bioactive compounds. One of the species that has been used in traditional medicine for a long time and with a valuable source of bioactive molecules is *Annona cherimola* Mill. (ACM) or the custard apple [1]. It belongs to the Annonaceae family, which comprises more than 140 genera and approximately 2500 species [2]. ACM is an extensively known deciduous tree whose edible fruit is known as the cherimoya. Cherimoyas are considered an exotic fruit native to inter-Andean valleys from Peru and Ecuador [3]. They can be found and are commercially cultivated in several mild-temperature regions around the world, such as Portugal, Italy, Taiwan or Spain [4]. Around 2406 hectares where mainly two varieties of this plant are cultivated, ‘Fino de Jete’ and ‘Campa’ [5] can be found in the coast of Granada-Malaga region (southern of Spain) also known as ‘Costa Tropical’ [6], which was granted by the European Union a Protected Designation of Origin (PDO) [7] in 2002. In fact, the specific soil and climate conditions as well as the particular requirements regarding the handling of a crop with a rather brief postharvest life [8] have made of Spain its world leading producer [2,9]. The commercialization of this cultivar from Coast of Granada-Malaga around the rest of the European regions has grown by 20% in the last year [10].

ACM is mainly cultivated for the food industry and although it is often consumed as fresh fruit in many countries, a varied range of food products and beverages are made up from cherimoya pulp [11]. In addition to its organoleptic properties and nutritional value, ACM has a certain potential use in folk medicine, particularly for the treatment of skin disorders [12]. Additionally, some hepatoprotective, anti-inflammatory and antitumoral properties have been described [1,2,9]. Over the last few decades, a growing number of studies have focused on the phytochemical composition of ACM extracts in order to explain their traditional applications and to determine the compounds in ACM that are responsible of its biological activity. Some phytochemicals, such as flavonoids, tocopherols, tannins, acetogenins, saponins, polyphenols among others, have been isolated from ACM roots, seeds, pulp and leaves [5,9,13]. Since the first acetogenin, uvaricin was isolated by Jolad et al. in 1982—a product of interest for its cytotoxic activity—and other investigations have focused on this class of compounds, which are exclusively found in the Annonaceae family [14]. Since then, over 500 acetogenins from the Annonaceae family have been described [15].

**Figure 1:**
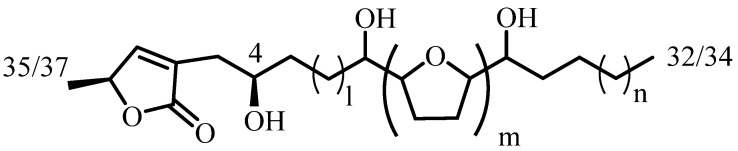
Overview of annonaceous ACG structure.

Annonaceous Acetogenins (ACGs) are waxy substances formed by derivatives of long-chain fatty acids where up to three THF rings, sometimes circled by hydroxyl groups, conform a polar core. Lineal ACGs, or with a central THP ring, double bonds and ketone groups, have also been described. One lactone ring, at one of the ends of the molecule, completes the structure up to C37 (Figure 1) [16,17,18,19].

ACGs are currently considered to be amongst the most potent antitumoral compounds [20]. Both in vitro and in vivo cytotoxic activity against a large number of tumoral cell lines—some of them resistant to regular therapies—have been studied [18,21,22]. These natural products obtained better results than those achieved by more conventional treatments. Given these properties, the extraction and purification of its individual compounds is a subject of interest. For this purpose, the use of bio-guided isolation conducted under chromatographically controlled procedures has been proposed. Such compounds of interest are often found in mixtures where content is scarce, which poses a difficulty for structure determination and extraction. The combined use of NMR and mass spectrometry is required for a definite identification. 

The number of bioactive compounds and their concentration levels may vary considerably according to the type of cultivar and its origin. 

## 2. Classification of ACGs from ACM

The most generally used classification for annonaceous ACGs is based on the structure of its systems of central rings. Hence, linear, mono-THF, adjacent or non-adjacent bis-THF, tri-THF, epoxy and THP ACGs, could be further divided attending to the number and location of the hydroxyl groups along the chain, and also according to the type of terminal lactone [15,18].

**Figure 2:**
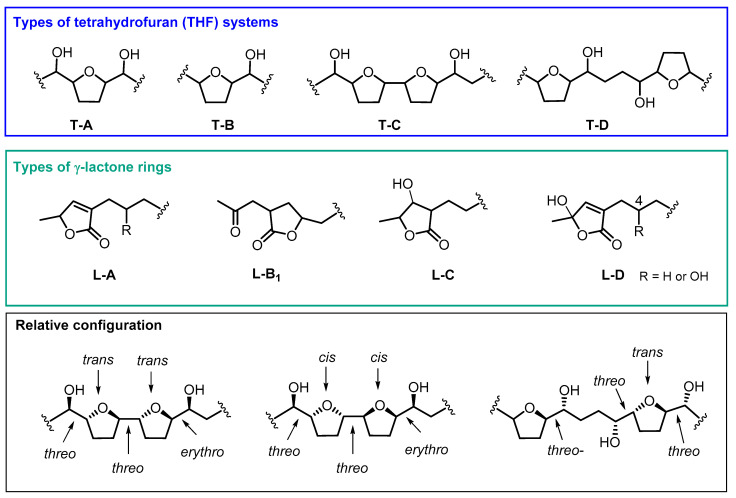
Most common structures of annonaceous acetogenins isolated from ACM.

Most of the ACGs found in ACM contain one or two THF rings. In addition, a number of variations have usually been registered with regard to the number and position of the hydroxyl groups. Between one and four hydroxyl groups can be found along the aliphatic chain or at the terminal lactone group. Moreover, some of the differences in the number and position of the hydroxyl groups, besides their stereochemistry, are crucial for their biological activity. The most common THF and γ-lactone ring structures can be seen in Figure 2. This classification has been made following the same criteria as Neske and cols [15].

**Figure 3:**
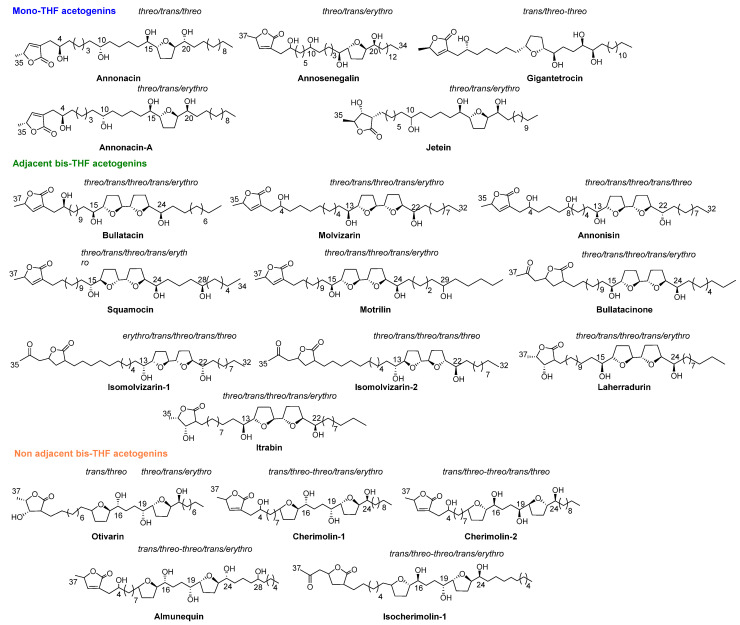
Annonaceous acetogenins isolated from ACMs harvested at ‘Tropical Coast’.

A total of 41 ACGs have been identified and isolated from roots, seeds, stems and, recently, from the deciduous leaves of ACM (Table 1). Of all these compounds, a total of 20 ACGs can be found in the ACMs from the Spanish ‘Tropical Coast’ region (Figure 3).

### 2.1. Mono THF Acetogenins

From 1990 to date, twenty-two ACGs from ACM have been described. This represents the main structural group of ACGs isolated from ACM and they have been mostly found as mono-THF α,α’-dihydroxylated ACGs (T-A). The most frequent configuration of the diastereoisomers is threo/trans/threo. Most of the mono THF acetogenins from ACM possess a α,β-unsaturated methyl γ-lactone ring (L-A).

### 2.2. Adjacent Bis-THF Acetogenins

This second structural classification constitutes the next most frequent group. Thirteen ACGs from ACM have been described as adjacent bis-THF acetogenins. This bis-THF system is found flanked at the α and α’ positions by two hydroxyl groups (T-C). Moreover, three types of γ-lactone moiety have been reported, including α,β-unsaturated methyl γ-lactone (L-A); acetonyl γ-lactone (L-B_1_) and β-hydroxyl methyl γ-lactone (L-C) (Table 1 and Figure 3). Nearly all of the ACGs in this class present a threo/trans/threo/trans/erythro configuration.

### 2.3. Non-Adjacent Bis-THF Acetogenins

This constitutes the minor group, with only six ACGs from ACM described until present; two of them display a hydroxyl group at C4, namely cherimolin-1 and cherimolin-2. Two types of γ-lactone systems, L-A and L-B_1_, have been described in this group.

## 3. Methods Applied for the Purification and Isolation of the ACGs Described in ACM

A wide variety of separation techniques have been successfully used to isolate even closely related ACG compounds [18]. Most methods comprise a first phase consisting of the extraction of the dried plant material using methanol, ethanol or dichloromethane as a solvent. Then, this first step is followed by further fractionations by means of different separating chromatographic techniques, including open-column chromatography (OPC), flash chromatography (FC), preparative thin-layer chromatography (PTLC) or high-performance liquid chromatography (HPLC) [37]. In some cases, the peculiar structure that these naturally occurring compounds present demand the use of rather complex eluent-solvent mixtures (Table 2).

Moreover, two methods have been used to monitor the fractionation of the ACGs: brine shrimp lethality test (BST) and Kedde’s reagent [37]. *Artemia salina*, commonly known as brine shrimp, is a small crustacean which has been the subject of many studies. The brine shrimp lethality test is an economic, rapid and reliable tool for the preliminary assessment of general toxicity [38]. This first method allows the determination of the most bioactive compounds in a particular extract, and it has rendered good results with acetogenins [39]. On the other hand, Kedde’s reagent is not specific for ACGs but it allows the detection of its unsaturated conjugated lactone moiety. This reagent consists of a solution of 3,5-dinitrobenzoic acid in ethanol (2% *w*/*v*) and potassium hydroxide in ethanol (5.7% *w*/*v*) mixed at equal volumes to produce a pink coloration in the sample solution when α,β-unsaturated γ-lactone moiety is present [39,40].

All in all, methanol is the most often preferred solvent for the extraction of ACGs. After the extraction, the crude extract is often partitioned using chloroform and water, since the chloroform layer becomes enriched with the ACGs. Owing to the structural similarities between the different ACGs, their isolation presents some difficulties. Thus, repeated open-column chromatography and high-performance liquid chromatography (HPLC) are often used for their isolation. 

## 4. Biological Activity and Anticarcinogenic Properties of the ACGs Isolated from ACM

ACGs have recently generated considerable interest because of the wide range of biological activities that they have exhibited, such as anti-inflammatory, pesticidal, antimicrobial and particularly cytotoxic activities. Several studies have already demonstrated that these compounds present either none or minimal toxicity to normal cells but are highly toxic to cancerous cells [20,40,41]. 

ACGs are currently considered to be amongst the most potent antitumoral compounds and are also effective for the treatment of drug-resistant cancerous cells. It has been well established that, among other modes of action, this type of compound inhibits the complex I (NADH-ubiquinone oxidoreductase) found in mitochondrial electron transport systems as well as glucose uptake (acting as potent modulators of glucose transporters) and also targets hypoxia-inducible factor-1 (HIF-1). The selectivity of ACGs regarding tumoral cells could be explained by the higher NADH oxidase content accompanied by the increased ATP demand that characterize tumoral cells [39,41]. Additionally, several studies have reported that the ACG’s functional group related to their antineoplastic activity seems to be their mono or bis-tetrahydrofuran ring accompanied by two or more hydroxyl groups [42]. Thus, the administration of adjacent bis-THF bullatacin, α,β-unsaturated methyl γ-lactone (L-A) ring at a dose of 15 μg/kg, reduced tumor growth in mice bearing S180 and HepS xenografts by 65.8% and 63.4%, respectively. These results were superior to those obtained using higher concentrations of taxol (40 μg/kg) [43]. Other studies have highlighted the cytotoxicity of laherradurin, which also possesses an adjacent bis-THF moiety (T-C) and β-hydroxyl methyl γ-lactone ring (L-C). This ACG has succeeded in reducing the size of HeLa tumors by similar values to those obtained when doxorubicin (antineoplasic drug) was employed. Likewise, cherimolin-2, an ACG that contains a non-adjacent bis-THF moiety (T-D) and a γ-lactone unit (L-A), also reduced the size of tumoral cells, even if it did not reach laherradurin’s effectiveness [42,44]. 

Annonacin, which contains a mono-THF α,α’-dihydroxylated system (T-A) and a α,β-unsaturated methyl γ-lactone ring (L-A), has been one of the most deeply studied ACGs in the last two decades. This ACG can be found in a large number of species from the Annonaceae family and good yields can be obtained through isolation processes. Numerous studies have highlighted the cytotoxicity of this mono-THF ACG. Thus, Yuan et al. have described that annonacin is able to activate p21 and arrested cancer cells at a growth-static G1 phase. Furthermore, it presented more significant cytotoxicity to cancer cells in growth (S phase cells) [45]. Other research by Roduan et al. demonstrated that the application of annonacin at 85 nM significantly reduced tumor incidence as well as volume in a two-stage mouse skin tumorigenesis model. Annonacin also appeared to be non-toxic to liver and kidney, which suggests its potential as a therapeutic compound for the prevention and treatment of skin cancer [46]. Wang et al. administered 10 mg/kg of annonacin orally to in vivo hybrid mice (BDF-1) models and successfully inhibit lung cancer by 57.9% [47]. 

Moreover, the pesticidal potential of these metabolites has also been reported. For instance, McLaughlin et al. performed a comparative structure–activity relationship (SAR) evaluation on the toxicity of 44 ACGs to yellow fever mosquito larvae. Their results revealed that the compounds bearing adjacent bis-THF rings with three hydroxyl groups were the most potent ones. Among the ACGs evaluated, bullatacin, with an LC_50_ value of 0.1 mg/L, showed the highest activity against yellow fever mosquito larvae. The compounds with LC_50_ values below 1.0 mg/L in this assay were considered to be potential candidates for the development of new pesticides [48].

According to other research studies, ACGs could be involved in the pathogenesis of certain neurodegenerative disorders, such as in the case of the high prevalence of atypical parkinsonism that occurs in Guadeloupe, in some parts of the Afro-Caribbean region and among the Indian population residing in London and New Caledonia. This could be partially explained by the high consumption of dietary supplements and fruit products containing plant material from Annonaceae. A study performed by Lannuzel et al. described that annonacin (extracted from graviola) was toxic to embryonic rat primary mesencephalic and striatal neurons [49]. Nevertheless, another study carried out by Potts et al. reported that the toxicity of annonacin was less potent to cortical neurons than previously reported to mesencephalic and striatal neurons [50]. Champy et al. investigated the content of annonacin (the major acetogenin) in extracts from fruits and leaves of *A. muricata*. It was estimated that a daily consumption of one piece of *A. muricata* fruit for one year would result in an aggregate amount of annonacin equivalent to the necessary dose of the purified compound to be administered by intravenous infusion to induce brain lesions in rats [51]. However, Awodele and co-workers demonstrated that the consumption of *A. muricata* fruit for 60 consecutive days did not induce any significant toxicity in rat models [52]. This neurotoxicity could also be increased by adding other metabolites, such as isoquinolinic alkaloids or through the synergic effect with other ACGs. Although evidence may still remain circumstantial, chronic toxicity as a result of regular Annonaceae consumption might pose a serious public health issue. Therefore, additional studies to determine the potential risks of neurodegenerative disorders associated with chronic exposure due to regular intake of Annonaceae products are needed. It is well known that any compound can be considered toxic, depending on its dose. It is, therefore, absolutely crucial to precisely determine the concentration of these metabolites as well as other chemical constituents in fruit products, infusions, nutritional supplements or decoctions made out of plants from the Annonaceae family. Likewise, further research on the mechanism of action supported by both in vitro and in vivo experiments are also required. Alternatively, these naturally occurring acetogenins could be modified with the aim of producing improved derivatives with a lesser toxicity level. 

## 5. How to Apply ACGs from *Annona Cherimola* Mill.

The acetogenins that can be found in *Annona cherimola* have been the center of attention of many researchers because of their cytotoxic effect in different cancer lines [53], as well as for their tumoral and mitochondrial complex I inhibitory capacities [32,54]. In addition, these natural products have also exhibited antiparasitic [48], insecticidal [55,56] and bactericide [57] activity with really promising results. 

Despite this wide array of possibilities, the isolation and identification of acetogenins still represent an obstacle in reaching their practical application. Nevertheless, certain counter current chromatographic techniques, together with a more recent method based on absorbent biopolymers, seem to provide more efficient ways to collect acetogenins [58,59,60]. Thanks to these new isolation methods, a growing number of studies on cell lines, isolated enzymes and complexes have been completed. Larger scale procedures, however, are still to overcome amount, solubility and bioavailability limitations. 

The formulations that are usually employed to test the effect of acetogenin from *Annona cherimola* on cell lines are mainly obtained with the use of organic solvents. Dimethyl sulphoxide is the most employed solvent in these tests, being applied either as co-solvent (1%) with water or as single solvent. Annonacin and molvizarin, obtained through this single solvent formulation, have been tested for larvicidal [56], anti-protist (leishmanicidal and trypanocidal) [61] and different insecticidal activities, such as oviposition capacity [13]. Apart from that, kidney studies with VERO cells have also been carried out on acetogenins combined with rollisniastatin-2 and on squamocin, where pure a DMSO solution was mixed with cell cultures [61,62]. Methanol and ethanol solutions have also been employed to test acetogenin obtained from *Annona cherimola*. Its insecticidal activity against mosquito and different lepidoptera larvae were assayed by He and co-workers [48]. Tolosa et al. [55] employed methanol to solve motrilin, rolliniastatin-2, bullatacin and annonacin. Mclaughlin’s research team [63,64] used ethanol solutions and the same acetogenins plus aromin-A to evaluate their effects on lung, breast and colon tumor cell lines. Hidalgo et al. [65] conducted a number of bioassays against lepidoptera larvae, where other lesser known *Annona cherimola* acetogenins, such as almunequin, cherimolin-1, cherimolin-2 and squamocin were tested, and acetone was used as an alternative solvent.

Notwithstanding the good results achieved by solving the acetogenins in organic solvents, this method is not feasible for further objectives, such as their application to human assays, since their inoculation while mixed in pure ethanol or DMSO is not an acceptable approach. This is one of the reasons why some authors have focused on alternative formulae that intend to compensate poor water solubility. In short, two methods mainly based on encapsulation techniques have been devised to improve the performance of *Annona cherimola* acetogenins. The first one uses polymeric compounds to produce core/shell structures that mask the lower physicochemical properties of the lactones. Polyethylene glycol (PEG) with certain modifications is the most commonly used polymeric compound, but also a monomethoxy derivative (mPEG), ε-caprolactone (PCL), poly (lactide-co-glycolide) (PLGA) or cholesterol (Chol) have also been tested. These polymeric nanoparticles (NPs) have been successfully tested for the treatment of different types of breast cancer. For instance, Wang’s group tested the effect of bullatacin encapsulated in PCL-PEG nanoparticles against a 4T1 cancer cell line as well as in vivo breast tumor-bearing mice. They obtained a high percentage of 20–60 nm-size encapsulated NPs (>70%) which demonstrated twice the ability to reduce tumor volume than free bullatacin [66]. In a patented expansion of their study, the same authors applied their encapsulation method to squamocin and applied the formula to HeLa cell lines and ex vivo HeLa tumor-bearing mice. Tumor volume was thereby reduced by 64.11%, in comparison to the 43.1% reduction achieved by free squamocin. This novel method exhibited up to 144 h of controlled release of the drug [67]. A similar method has been patented by Ao et al. to apply the use of polymeric nanoparticles to cherimolin and motrilin for oral administration instead of being injected through the mice tail vein [68].

The same authors have also patented a method to modify the different polymers that had been previously assayed so that they would include different stabilizers, such as albumin serum or soybean lecithin, in the nanoparticle suspension. This new method was employed to encapsulate bullatacin, but they exhibited a shorter delivery time (controlled release for 96 h) compared to when just the polymer nanoparticles were administered. Nevertheless, this method seems to improve the antitumor efficacy of bullatacin against liver tumor, with a maximum inhibition rate of 74.8% in the tumor volume [69]. The size of the nanoparticles used in this method was similar to that of nanoparticles which had not been added a stabilizer, which means that these co-polymers did not affect the chemical structure layout [70]. 

**Figure 4:**
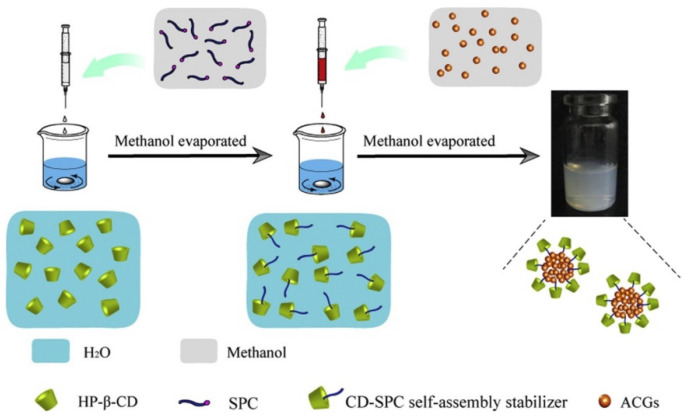
Diagram of the SMPMs synthesis to encapsulate *Annona cherimolia* acetogenins. (Reproduced from 10.1016/j.colsurfb.2016.05.012 with permission from Elsevier B.V. under License Number: 5039230526812) [75].

In a different line of investigation, micelles based on pseudorotaxanes with cyclodextrins (CDs) are becoming a prominent approach for the encapsulation of *Annona cherimola* acetogenins. Wang’s working group has used supramolecular polymer micelles (SMPMs) with β-cyclodextrin and 2-hydroxypropyl-β-cyclodextrin (HP-β-CD) to encapsulate bullatacin (Figure 4). One of the methods used employed folic acid as the guest molecule to be hosted in the cyclodextrin toroid, which would act as a building-block for the micelle, while another method employed soybean lecithin, a really standardized guest molecule in SMPMs science [71,72]. The nanostructures generated with the cyclodextrin molecules seem to be bigger than the particles synthesized using polymers, with sizes between 140 and 200 nm. In addition, the encapsulation percentages are also lower, with values between 45% and 60%. Despite these apparently worse results, this method allows for the incorporation of oligoelements, thanks to which it presents the advantage of serving a double purpose: anti-tumoral agent and nutraceutical supplier. In the case of the β-CD/folic acid micelles, the structure includes vitamin B_9_, which facilitates bio-recognition and targeting towards the liver. Besides from everything mentioned above, natural CDs are chemical compounds approved as a food additive by the EU since 2018, which supports their use as an excipient instead of other substances [73]. With respect to the inhibition of 4T1 tumors volume, there are no significant differences between the two methods, when compared against polymeric nanoparticles, with inhibition values at around 70%. It should be noted that drug release times change drastically as the micelles’ composition changes. In the case of the β-CD/folic acid, the drug release times are quite similar to those of polymeric nanoparticles, with 142 h required for the total delivery of the drug [74]. On the other hand, HP-β-CD/Lecithin presents an 80 h release profile [75]. The currently published papers do not cast any light over this behavior, but it could be attributed to the higher solubility of HP-β-CD in water when compared to that of β-CD. This improved solubility of the micelles in the media would facilitate their degradation. A similar method has been patented by Hong et al., where SMPMs are synthesized by means of three natural cyclodextrins (α, β and γ) that act as guests for different molecular weight polyethylene-glycol units ranging from 600 to 2000. No specific values have been reported on encapsulation percentages, although over 50% has been claimed. It should also be noted that bullatacin release time is limited to 72 h. When the different methods are applied to the same acetogenin and their data (Table 3) are compared, as in case of bullatacin, it seems that the guest molecule in the SMPMs building-block is crucial with respect to core release times, and their increment in molecular weight/molecular volume reduce the drug delivery time.

Gutiérrez et al. employed SMPMs based on α-CD and urea to encapsulate annonacin. In line with other studies, cytotoxicity to liver cells was the main objective, and it was proven that annonacin is selective to tumor cells, with over 87% of cell survival when the acetogenin is applied to non-tumoral liver cells (HEK-293) [20]. In this case, although both the encapsulation percentage and the drug delivery time provided the lowest values, it should be highlighted that annonacin is completely different from other previously encapsulated and tested acetogenins, since it presents a mono-THF structure instead of the bis-THF structure of bullatacin. No studies on the subject have been found to allow the comparison between these two methods.

Most of the formulation cases have focused on bullatacin, the most abundant acetogenin, but it should be emphasized that the authors used an acetogenin enrichment fraction and based their studies on determining bullatacin content, since it is the main compound in the fraction. However, an efficient encapsulation procedure for single acetogenins should be developed, similarly to the one previously described for anonacin, so that any antitumoral efficacy can be attributed to the only compound in the media and not to the possible synergistic effect that the different mixtures of acetogenins may exhibit.

## 6. *Annona Cherimola* Mill. Acetogenin Derivatives

From the moment acetogenins were discovered, many researchers have tried to synthesize mimics or analogous structures with a similar bioactivity profile but with a less complicated production process at laboratory scale. Attending to isolation yields, most authors reported values between 0.00019% and 0.003% [20,26], which poses a limit on the prospective practical usage of the acetogenins found in *Annona cherimola*. Naturally, different methods have been implemented to try and increase the yields obtained from the synthesis of acetogenins. One of such methods consists of modifying their THF core by incorporating a different functional group or structure that would facilitate the synthesis process. Miyoshi’s research team replaced the bis-THF core in bullatacin with either an ether group or a tetrahydroxyl core. For the first strategy, a 1,2-dimethoxyciclopentane derivative was used as the central unit, while for the second approach, a hexane-tetraol unit was employed. Both approaches were tested against NADH oxidase inhibition, but neither of them surpassed the activity level exhibited by bullatacin [77]. The dimethoxiciclopentane acetogenin mimic achieved comparable values (IC_50_ = 1.0 nM) with regard to bullatacine (IC_50_ = 0.83 nM) [78]. Nevertheless, the tetraol structure and the MOM protecting derivative are far from the expected values, with 20 nM and 4100 nM of IC_50_, respectively. Rodier et al. [79] employed a similar approach using linear ether groups to substitute the bis-THF core and obtain a bullatacinone mimic. In this case, it was tested against L1210 leukemia cells. The dimethoxiheptane acetogenin mimic achieved similar IC_50_ values to those of natural bullatacinone, which, considering the lower number of steps that are required for its synthesis, make this mimic a prominent candidate for further studies. 

**Figure 5:**
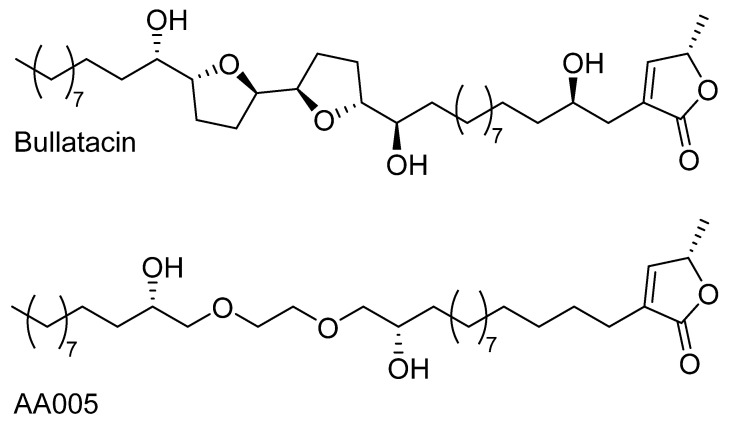
Structures of bullatacin and AA005.

Following the same approach as described above for lineal ethers, Yu et al have recently synthesized at gram-scale a new and interesting derivative, based on bullatacin (Figure 5), known as AA005, [80]. This *Annona cherimola* acetogenin mimic has been tested against human colon carcinoma cell line SW620 in vivo, and against different human cancer cell lines in vitro [81]. In the case of colon cancer, the anticancer effect goes through the down regulation of myeloid cell leukemia-1 protein, which is usually overexpressed or amplified in human cancer, which suggests its critical role in tumor cell survival [82]. Furthermore, according to the fluorescent images obtained from AA005-fluorescein derivatives that were administered into any of the hydroxyl positions [83], the AA005 compound presented a different distribution in healthy human cells compared to the accumulations on the mitochondria that only took place in cancerous cells. 

The same bullatacin mimic synthesis method has been used to incorporate more carbon-spacing units between the oxygen-ether atoms that are found in the core, but no higher inhibition values against ovarian cancer (KB), liver cancer (BEL-7402) or colon cancer cell lines (HT-29 and HCT-8) were expressed [84]. Based on these results, we can conclude that the core composition in *Annona cherimola* acetogenin mimics is highly relevant for an efficient inhibitory capacity. 

Following this line of investigation consisting of acetogenin core modification, Gould et al. synthesized a squamocin mimic. However, the original squamocin molecule was drastically denatured, since it had its characteristic γ-butyrolactone final ring and the ether groups removed. The most remarkable point in the mimic structure produced by Gould is the tetrahidropyrane core connected to the triazole ring, that replaces the bis-THF of squamocin [85]. Similar approaches were implemented by Ichimaru et al., where bullatacin mimics were synthesized employing piperazine core. However, the studies on competitive inhibition showed that the core modification led to a different mechanism of action, even though the mitochondrial complex I remained the target [86]. One of the interesting novelties in the synthesis method described above is the incorporation of an aryloxy group at the end of the chain instead of γ-butyrolactone, something that had been previously tested on both sides of the chain by the same research group, which synthesized a bullatacin mimic structure without relevant results with regard to the inhibition of the mitochondrial complex I [87]. On the other hand, the introduction of biotin instead of aryloxy groups exhibited comparable IC_50_ inhibition values of the same mitochondrial complex, with 3.9 nM of the mimic versus 1.8 nM of the actual bullatacin [88].

**Figure 6:**
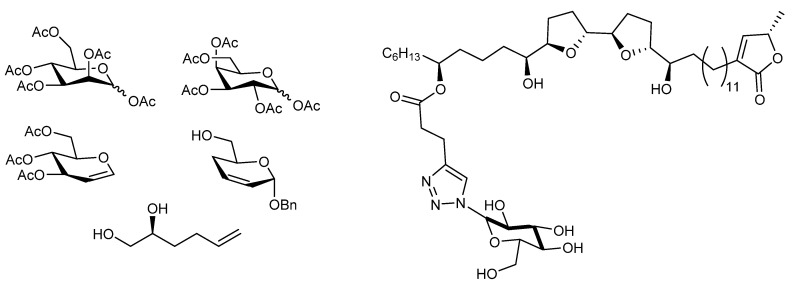
Carbohydrate precursors employed in the synthesis of *Annona cherimola* acetogenin mimics and mono-galactosylated squamocin.

Sugar residues have also been employed to replace THF segments, and Bachan et al. demonstrated that these bullatacin mimics present similar mechanism of action to real bullatacin when tested against HeLa, breast cancer (MDA-MB231), leukemia (Jurkat) and prostate cell lines (PC-3). Each one of the carbohydrate units tested were α-mannose derivatives with their hydroxyl groups replaced at different degrees. The units tested are presented in Figure 6. The bioactivity results are comparable to those obtained when using pure bullatacin, but with the advantage of improved water solubility [87]. Sugars have been recently introduced as a replacement of the hydroxyl groups in squamocin and bullatacin. Glucose or galactose units have been employed to generated acetogenin mimics with different substitution degrees. Among the derivatives synthesized, mono-galactosylated squamocin exhibited 1.37 mg/mL water solubility, while pure squamocin is totally insoluble in water.

Delving into the substitution of the γ-butyrolactone ring, Yabunaka and co-workers confirmed that the replacement of this group by an ubiquinone ring does not affect the inhibitory action of acetogenin. Inspired by previous experiences with bullatacin, they modified the chain end group by incorporating an ubiquinone ring that displayed the same inhibitory activity against mitochondrial complex I as the acetogenin original molecule [88,89].

In those cases, where the hydroxyl groups were replaced, similarly to in the previously mentioned experiments with sugars, the formation of acetyl and methoxymethyl (MOM) groups in squamocin, molvizarin, and motrilin was tested, but none of them exhibited an increment in their bioactivity when their insecticidal properties were assessed against the Spodoptera frugiperda larvae that infect corn and cotton crops [65]. On the other hand, the work developed by Shi et al. achieved more remarkable results when adding biotin units to squamocin and bullatacin to form acyl groups plus a spacer, instead of the hydroxyl groups. Bis-biotynilated squamocin with an ε-aminocapric acid spacer achieves better inhibition results against breast and mastocytoma cancer cell lines (4T1 and P815, respectively), if compared to those obtained when using pure bullatacin or squamocin. The synthesis of the bis-biotynilated squamocin can be completed in just two steps, but yields are quite low, in the order of between 1% and 20% [90].

We could conclude that the addition of biologically relevant structures (sugars or biotins) to *Annona cherimola* acetogenins through the functionalization of their hydroxyl group is a really good option to achieve an improved bioactivity. Even more remarkable is the enhancement of the physicochemical properties of acetogenins when certain carbohydrates are added. Unfortunately, this step requires the previous isolation of acetogenin, which poses a considerably limiting factor that could be overcome by implementing acetogenin mimics. Among such acetogenin mimics, AA005 stands out because it can be synthesized at gram-scale and also for having demonstrated its anticancer effect through in vivo studies.

## 7. Summary and Perspectives

Annonaceous acetogenins (ACGs) are one of the most interesting plant-derived natural compounds. They constitute a unique class of C35 or C37 secondary metabolites derived from the polyketide pathway. Since the first report on the bioactivity of uvaricin in 1982, isolated from the roots of Uvaria accuminata Oliv. by Jolad et al., which exhibited excellent bioactivity in the P-388 lymphocytic leukemia system in mice, these natural products have been extensively investigated. Moreover, they are recognized as one of the most powerful groups of complex inhibitors. These metabolites have shown a wide spectrum of biological activities, with particular reference to their anticarcinogenic properties. It has been demonstrated that certain variations in their backbone, including their THF system, the type of terminal lactone group, the number and position of their hydroxyl groups and their stereochemistry are crucial factors that influence their biological activity. It has been generally confirmed by most studies that ACGs containing adjacent bis-THF (T-C) and α,β-unsaturated methyl γ-lactone (L-A) ring were more active than those with non-adjacent groups or with the β-hydroxyl methyl γ-lactone group (L-C) [15]. However, further studies on the correlation between their structure and their activity are needed in order to clearly determine this association between structural requirements and biological activity.

Recent studies have further delved into novel formulation methods, other than the usual co-solvent, to be successfully applied to ACGs. Among them, two methods have gathered special attention, namely, polymeric nanoparticles (NPs) and micelle synthesis (SMPMs). Both of them have rendered promising results regarding the modulation of ACGs delivery, their stability and their water solubility. 

Given that *Annona cherimola* Mill acetogenins are generally found in low concentration mixtures, a deeper knowledge on such structural requirements should lead to the desired development of new synthetic derivatives and more efficient drugs. In this line, a number of studies have focused on the synthesis of mimics and derivatives, but none of these has surpassed the cytotoxic and antitumor profiles already exhibited by the natural single compounds. Nevertheless, AA005, a mimic of bullatacin, has stood out because of the possibility of synthesizing it at gram-scale and also for its good in vivo results; two factors that make it a promising compound. Furthermore, the modification of natural acetogenin to incorporate sugar units seems to open the way to increasing the solubility of these compounds in water, while conveniently enhancing some of its physicochemical properties. Despite the ancient and popular consumption of the fresh fruit and other food products derived from these plants, further studies would be required to determine safe consumption doses as well as to develop an efficient isolation and administration methodology that allows for the benefit of an efficient medical use of their recognized bioactivity.

## Figures and Tables

**Table 1:** Characteristics and structural features of the 41 ACGs described in ACM.

CAS Number	Name	Other Names	OH Positions	THF System	Relative THF Configuration	Type of γ-Lactone Ring	Molecular Formula	Organ	Number of Biological Studies (Scifinder Database)	Ref.
133352-34-8	Corossolin	Corossoline	10,15,20	T-A	*th/t/th*	L-A	C_35_H_64_O_6_	Seeds	15	[23,24]
246165-35-5	Annocherin		4,15,20	T-A	*th/t/th*	L-A	C_35_H_62_O_7_	Seeds	2	[25]
111035-65-5	Annonacin ^†^		4,10,15,20	T-A	*th/t/th*	L-A	C_35_H_64_O_7_	Seeds, deciduous leaves	133	[20,26]
130853-76-8	Annonacin-A ^†^		4,10,15,20	T-A	*th/t/er*	L-A	C_35_H_64_O_7_	Seeds	17	[27]
137550-92-6	Annomontacin		4,10,17,22	T-A	*th/t/th*	L-A	C_37_H_68_O_7_	Seeds	18	[26]
155969-86-1	Xylomaticin		4,10,15,20	T-A	*th/t/th*	L-A	C_37_H_68_O_7_	Seeds	7	[23]
155969-65-6	Gonionenin	(2,4-*cis*)-Gonioneninone	4,10,13,18	T-A	*th/t/th*	L-A	C_37_H_66_O_7_	Seeds	8	[23]
176200-77-4	Annosenegalin ^†^		4,10,15,20	T-A	*th/t/er*	L-A	C_37_H_68_O_7_	Seeds	1	[27]
172586-13-9	*Cis* annonacin		4,10,15,20	T-A	*th/c/th*	L-A	C_35_H_64_O_7_	Seeds	10	[28]
344940-10-9	Annocherimolin		4,9,13,18	T-A	*th/t/th*	L-A	C_37_H_66_O_7_	Seeds	1	[29]
373362-55-1	Annomocherin		4,10,15,20	T-A	*th/t/th*	L-A	C_35_H_62_O_7_	Seeds	1	[26]
134955-48-9	Gigantetrocin ^†^		4,14,17,18	T-B	*t/th-th*	L-A	C_35_H_64_O_7_	Seeds	13	[27]
344940-09-6	Annomolin		4,7,8,18	T-B	*th-t/th*	L-A	C_35_H_64_O_7_	Seeds	1	[29]
152784-18-4 and 152784-19-5	*cis*/*trans* isoannonacins	*cis*-Annonacin-A-one and *trans*-Annonacin-A-one	10,15,20	T-A	*th/t/th*	L-B_1_	C_35_H_64_O_7_	Seeds	7	[28]
246165-37-7 and 246165-38-8	(2,4)-*cis*- and *trans* annocherinones		15,20	T-A	*th/t/th*	L-B_1_	C_35_H_62_O_7_	Seeds	2	[25]
627518-99-4 and 627519-01-1	Annomolon-A + 34-epi		15,20,34	T-A	*th/t/th*	L-D	C_35_H_62_O_7_	Seeds	1	[30]
627519-00-0 and 627519-02-2	Annomolon-B + 34-epi		4,15,20,34	T-A	*th/t/th*	L-D	C_35_H_62_O_8_	Seeds	1	[30]
139294-55-6	Jetein ^†^		10,15,20	T-A	*th/t/er*	L-C	C_35_H_66_O_7_	Seeds	2	[31]
102989-24-2	Asimicin	Squamocin H	4,15,24	T-C	*th/t/th/t/th*	L-A	C_37_H_66_O_7_	Seeds	57	[32]
123123-32-0	Bullatacin ^†^	Annonareticin; LI 12105; Rolliniastatin 2; Squamocin G	4,15,24	T-C	*th/t/th/t/er*	L-A	C_37_H_66_O_7_	Seeds	229	[31]
138551-26-5	Molvizarin ^†^		4,13,22	T-C	*th/t/th/t/er*	L-A	C_35_H_62_O_7_	Seeds, deciduous leaves	17	[20,33]
194413-43-9	Annonisin ^†^		4,8,13,22	T-C	*th/t/th/t/th*	L-A	C_35_H_62_O_8_	Deciduous leaves	3	[20]
120298-30-8	Squamocin ^†^	Annonin I; Squamocin A	15,24,28	T-C	*th/t/th/t/er*	L-A	C_37_H_66_O_7_	Seeds, roots	162	[31,34]
138551-27-6	Motrilin ^†^		15,24,29	T-C	*th/t/th/t/er*	L-A	C_37_H_66_O_7_	Seeds, deciduous leaves	28	[20,33]
159934-23-3	Squamocin B		13,22,26	T-C	*th/t/th/t/er*	L-A	C_35_H_62_O_7_	Seeds	10	[32]
123012-00-0	Bullatacinone ^†^	Isorolliniastatin-2	15,24	T-C	*th/t/th/t/er*	L-B_1_	C_37_H_66_O_7_	Roots	69	[34]
161169-72-8	Isomolvizarin-1 ^†^		13,22	T-C	*th/t/th/t/er*	L-B_1_	C_35_H_62_O_7_	Roots	0	[34]
158252-75-6	Isomolvizarin-2 ^†^		13,22	T-C	*th/t/th/t/th*	L-B_1_	C_35_H_62_O_7_	Roots	1	[34]
125276-75-7	Laherradurin ^†^		15,24,35	T-C	*th/t/th/t/er*	L-C	C_37_H_68_O_7_	Seeds	13	[35]
139294-54-5	Itrabin ^†^		13,22,33	T-C	*th/t/th/t/er*	L-C	C_35_H_64_O_7_	Seeds	8	[33]
832683-48-4	Tucumanin		15,24,35	T-C	*th/t/th/t/th*	L-C	C_37_H_68_O_7_	Seeds	3	[32]
92280-15-4	Otivarin ^†^		16,19,24,35	T-D	*t/th-th/t/er*	L-C	C_37_H_68_O_8_	Seeds	6	[31]
92280-14-3	Cherimolin-1 ^†^	Bullatalicin	4,16,19,24	T-D	*t/th-th/t/er*	L-A	C_37_H_66_O_8_	Seeds, deciduous leaves	16	[20,31]
151637-38-6	Cherimolin-2 ^†^	Bullatanocin	4,16,19,24	T-D	*t/th-th/t/th*	L-A	C_37_H_66_O_8_	Seeds	10	[31]
125620-82-8	Almunequin ^†^	Squamostatin-A	16,19,24,28	T-D	*t/th-th/t/er*	L-A	C_37_H_66_O_8_	Seeds, roots	12	[31,34]
241822-07-1	Aromin-A		15,20	T-D	*t-th/t/er*	L-A	C_35_H_60_O_7_	Stems	1	[36]
157966-80-8	Isocherimolin-1 ^†^		16,19,24	T-D	*th-th/t/er*	L-B_1_	C_37_H_66_O_8_	Roots	2	[34]

^†^ ACM from the Spanish ‘Tropical Coast’; *th* = *threo*; *er* = *erythro*; *t* = *trans*; *c* = *cis*.

**Table 2:** ACGs isolated from *A. cherimola* Mill.

Method	Eluent System	Isolated ACGs	Organ	Reference
Flash chromatography	CH_2_Cl_2_-EtOAc-MeOH (4:14:1)	Molvizarin ^†^, motrilin ^†^, bullatacin ^†^, squamocin ^†^, cherimolin-1 ^†^, cherimolin-2 ^†^, almunequin ^†^, itrabin ^†^, laherradurin ^†^, otivarin ^†^, jetein ^†^	Seeds	[31,33]
Flash chromatography	CH_2_Cl_2_-EtOAc-MeOH (20:19:1) and CH_2_Cl_2_-MeOH (24:1)	Annosenegalin ^†^, annonacin-A ^†^, gigantetrocin ^†^	Seeds	[27]
Open column chromatography HPLC	MeOH-Hexane (1:1) ACN-H_2_O (80:20)	Annomolin ^‡^, Annocherimolin ^‡^	Seeds	[29]
Open column chromatography HPLC	Hexane-CHCl_3_ and CHCl_3_-MeOH gradients ACN-H_2_O (80:20)	Annomolon A + 34-*epi*-annomolon A ^‡^, annomolon B + 34-*epi*-annomolon B ^‡^	Seeds	[30]
Open column chromatography HPLC	Hexane-CHCl_3_-MeOH gradient ACN-H_2_O (85:15)	Annocherin ^‡^, (2,4)-*cis*- and *trans*-annocherinones ^‡^	Seeds	[25]
Open column chromatography HPLC	Hexane-EtOAc gradient CH_2_Cl_2_-MeOH (95:5) MeOH-H_2_O-THF (80:20:5)	Molvizarin ^§^, motrilin ^§^, bullatacin ^§^, tucumanin ^§^, squamocin ^§^, squamocin B ^§^, laherradurin ^§^, itrabin ^§^, cherimolin-1 ^§^, almunequin ^§^, asimicin ^§^	Seeds	[32]
Open column chromatography HPLC	Hexane-CHCl_3_-MeOH gradient ACN- H_2_O (85:15)	*cis*-Annonacin ^‡^ and (2,4)-*cis*- and *trans*-lsoannonacins ^‡^	Seeds	[28]
Open column chromatography HPLC	Hexane-CHCl_3_-MeOH gradient ACN-H_2_O (85:15)	Annomocherin ^‡^, annonacin ^‡^, annomontacin ^‡^	Seeds	[26]
Open column chromatography HPLC	Hexane-CHCl_3_-MeOH gradient ACN-H_2_O (85:15)	Xylomaticin ^‡^, gonionenin ^‡^	Seeds	[23]
Open column chromatography PTLC	Hexane-acetone gradient EtOAc-hexane-acetone (10:10:1) EtOAc-acetone (10:1) CHCl_3_-MeOH (10:1) EtOAc-acetone (15:1)	Aromin-A ^#^, squamocin ^#^	Stems	[36]
Open column chromatography HPLC	CH_2_Cl_2_-MeOH (97:3) ACN-H_2_O-THF (70:30:2)	Isocherimolin-1 ^†^, isomolvizarin-1 ^†^, isomolvizarin-2 ^†^, bullatacinone ^†^, squamocin ^†^, almunequin ^†^	Roots	[34]
Vacuum column chromatography Open column chromatography HPLC PTLC	H_2_O-MeOH gradient EtOAc-Hexane gradient Hexane-EtOAc-acetone (7:2:1) CHCl_3_-MeOH-acetone-H_2_O (13:7:1:3)	Molvizarin ^†^, cherimolin-1 ^†^, motrilin ^†^, annonacin ^†^, annonisin ^†^	Deciduous leaves	[20]

^†^ ACM from the Spanish ‘Tropical Coast’. ^‡^ ACM from California. ^§^ ACM from Argentina. ^#^ ACM from Taiwan.

**Table 3:** SMPMs: Reported values of acetogenins.

Method	*Annona cherimola* ACG	Release Time (h)	Encapsulation (%)	M. W. Guest (g/mol)	Ref.
β-CD/Folic Acid	Bullatacin	142	58	441.14	[74]
HP-β-CD/Lecithin	Bullatacin	80	46	643.90	[75]
β-CD/PEG	Bullatacin	72	50	600.00–2000.00	[76]
α-CD/Urea	Annonacin	33	35	60.02	[20]
